# Effect of scanner lens on lateral response artefact in radiochromic film dosimetry

**DOI:** 10.1007/s13246-022-01136-0

**Published:** 2022-05-30

**Authors:** Tarafder Shameem, Nick Bennie, Martin Butson, David Thwaites

**Affiliations:** 1 North Coast Cancer Institute, Lismore, NSW Australia; 2 Institute of Medical Physics, School of Physics, University of Sydney, Sydney, NSW Australia; 3 EPA, Sydney, NSW Australia

**Keywords:** Radiotherapy dosimetry, Radiochromic film, Gafchromic, Epson scanner, Film dosimetry, Lens effect

## Abstract

Radiochromic film is a good dosimeter choice for patient QA for complex treatment techniques because of its near tissue equivalency, high spatial resolution and established method of use. Commercial scanners are typically used for film dosimetry, with Epson scanners being the most common. Radiochromic film dosimetry is not straightforward having some well-defined problems which must be considered, one of the main ones being the Lateral Response Artefact (LRA) effect. Previous studies showed that the contributing factors to LRA are from the structure of the active ingredients of the film and the components and construction of the flatbed scanner. This study investigated the effect of the scanner lens on the LRA effect, as part of a wider investigation of scanner design effects and uncertainties. Gafchromic EBT3 films were irradiated with 40 × 40 cm^2^ field size 6 MV beams. Films were analysed using images captured by a Canon 7D camera utilising 18 mm, 50 mm and 100 mm focal length lenses compared to images scanned with a conventional Epson V700 scanner. The magnitude of the LRA was observed to be dependent on the focal length of the lens used to image the film. A substantial reduction in LRA was seen with the use of the 50 mm and 100 mm lenses, by factors of 3–5 for the 50 mm lens and 4–30 for the 100 mm lens compared to conventional desktop scanner techniques. This is expected to be from the longer focal length camera lens system being able to collect more light from distant areas compared to the scanner-based system. This provides an opportunity to design film dosimetry systems that minimise this artefact.

## Introduction

Complex radiotherapy treatment techniques (IMRT, VMAT, SABR, SBRT) need patient-specific QA to check dose delivery and target volume localisation in three dimensions. Radiochromic film is a good dosimeter of choice for this purpose because of its properties of near tissue equivalency, very high spatial resolution and established method of use [1–5]. Like other dosimeters available it also has some drawbacks. Two main issues associated with radiochromic film dosimetry are the orientation effect and the lateral response artefact (LRA) effect [5–13], both apparent in scanning the films using the usual approach based on commercial flatbed scanners, where Epson scanners are the most commonly used. The orientation effect is defined as the change of response of radiochromic film depending on the orientation of the film on the scanner bed and the LRA effect is the change of response from middle to side of the film, orthogonal to the scanner’s light source travel direction [1, 6, 9, 13–15]. The orientation effect can be minimised with a strict protocol of marking and placing the film in the same orientation throughout the process. Hence the LRA effect remains as a main issue which has been investigated widely [10, 16–20]. The magnitude of light polarization, introduced by the scanner and the film, increases with increasing lateral distance from the centre of the scanner [11]. The size of the LRA effect depends on irradiated dose and position of the film on the scanner bed [11, 14, 16]. One contributor to the LRA effect is the needle-like crystal structure [9, 21, 22] in the active layer of radiochromic films. The rod- or hair-like crystals contribute to polarisation and anisotropic light scattering [23]. Upon irradiation, the neighbouring polymers create bonds and turn into even longer rods which enhances both of these phenomena. The other contributor is the scanner itself, e.g. from lens, mirror system and scanner bed [12]. Schoenfeld [9] showed a schematic diagram of the mirror system which shows the light travel path from light source to the lens system and CCD imager. The different scanner components contribute to the LRA effect [11, 14, 16] in different ways. Wide angle lenses and a mirror system [9] are used in Epson scanners to make them compact. However, wide angle lenses fail to collect all the light. In addition, these components and the scanner bed [12] add extra light polarisation. To manage the LRA effect, a correction factor is needed.

The purpose of this work is to investigate a novel technique using cameras and different lenses for radiochromic film imaging and analysis to evaluate the effect of focal length of the scanner lens on the LRA effect in radiochromic film dosimetry and to consider whether LRA effects, and correction factors, could be reduced by using a different lens system. This is part of wider investigations considering each component of the scanning system, aiming to explore the potential for a more optimised design for film dosimetry.

## Method

**Fig. 1:**
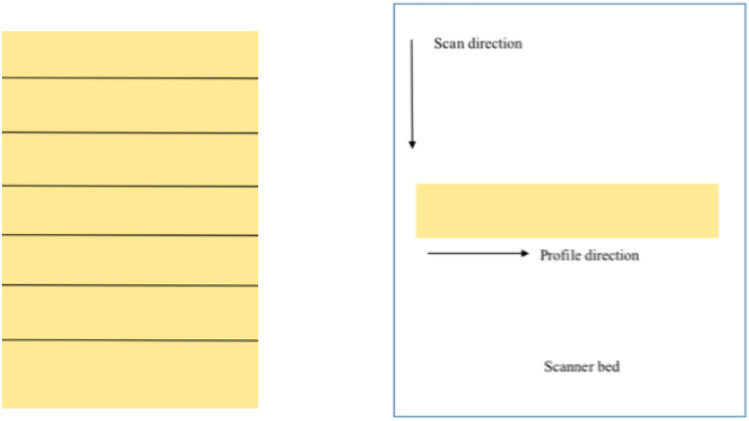
A representative schematic diagram showing the manner in which the films were cut, the orientation of the film pieces with respect to scan direction and the profile direction

The film preparation, handling and irradiation methods were similar to those described in previous work [24], but essential detail is repeated here for completeness. EBT3 films were cut into 3 cm × 20.3 cm strips along the short side of the film. Figure 1 shows schematically how the films were cut and the orientation of the film pieces on the scanner bed. The light source of the scanner is across the short side of the scanner bed, which means the longer side of a film strip on the scanner bed is parallel to the light source. This orientation of film pieces with respect to the light source was kept the same when images were taken with the camera. Films were irradiated at 10 cm depth of plastic water with 10 cm backscatter in a phantom that was 30 cm × 30 cm area presented to large area beams to achieve uniform dose across the film. Film pieces were irradiated individually for 100 MU, 200 MU, 500 MU and 1000 MU on an Elekta Synergy linear accelerator (linac) using a 6 MV beam and a 40 cm × 40 cm field size, giving doses to the film of 1.13 Gy, 2.25 Gy, 5.64 Gy and 11.28 Gy respectively.

Different studies have used different time, ranging from hours to days, for leaving the film in the box before scanning. Roozen [25] stated that 2–3 h is sufficient to stabilise the response of EBT film, referring to information from the manufacturer. Rink [26] presented a graph of time versus change of OD, showing insignificant change after 2 h. In this study, irradiated EBT3 films were left in the box for 2 h and then scanned using an Epson V700 [24]. The cut film pieces were taped down to the scanner bed to flatten the curvature. In addition, photos were taken with a Canon 7D DSLR camera. Gloves were used all the time during handling the film to avoid any contamination from fingerprint marks. The following settings were used for the scanner

- Mode: Professional
- Document type: Film (with film area guide); this setting allows transmission scanning
- Film type: Positive film
- Image type: 48-bit colour
- Resolution: 508 dpi
- No colour correction was applied

The following settings were used for the Canon 7D camera

- ISO: 100
- Shutter speed; 1/500
- Aperture: f4.5
- The room light was on
- No colour correction was applied when converting the RAW image to.tiff image

The lens system of an Epson V700 scanner has two lenses; one has a larger diameter than the other. The smaller one is for high resolution scanning. The larger lens is used for the scanning mode and so is the one used in this work. The lens assembly (both the lenses) was taken out and the focal length measured, by putting it against a vertical steel ruler on the floor on a piece of paper and moving it vertically to get a sharp image of a ceiling light. The distance from the image to the bottom lens is the effective focal length of the lens system. The focal lengths of both the lenses were found to be the same. The focal lengths of the Canon camera lenses were also verified in the same manner.

Previous studies have used a variety of methods to quantify the LRA effect, all of which are based on the difference in pixel values between the centre and a lateral position [9–12]. In this study, the LRA effect is represented as the maximum percent difference of average pixel values of 25 data points at both ends from the average of the central 25 data points.

### Scanning with an Epson V700 scanner

The scanning area, which is smaller than the scanner bed of the Epson V700 scanner is the same as an A4 document size. The short side of EBT3 film is also the same as A4 document size. The edges of the film strip therefore match the edge of the scanning area in the measured profile direction. The film pieces were placed at the central position on the scanner bed to ensure the whole film piece is in the scanning area. Each film piece was scanned 20 times. The scanned images were saved as *.tiff (tagged image file format) which were read and separated into three colour channels in ImageJ V1.49 software.

### Photos with a DSLR camera

**Fig. 2:**
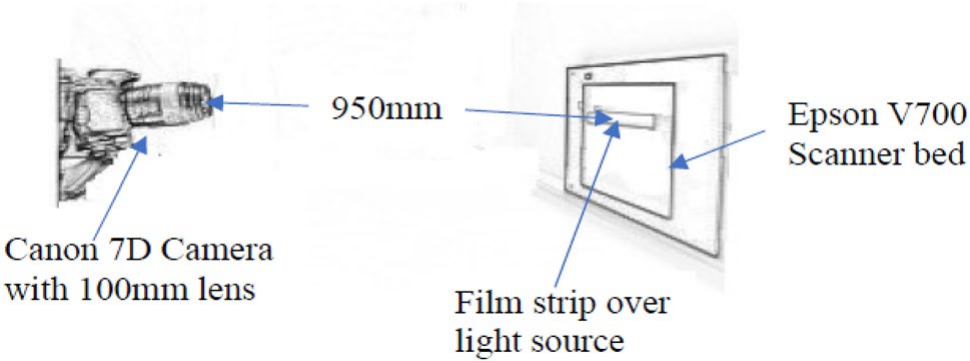
Camera set up for taking photos of film strips. This schematic example shows the set up for the 100 mm lens, with a distance between lens and film of 950 mm. The distances for the other lenses were 150 mm and 500 mm for the 18 mm and 50 mm lenses respectively

A LED light source, with a similar range of wavelengths as the Epson scanner light source, was wrapped with a diffuser and positioned on a wall. A V700 scanner bed was placed in front of the LED light source to keep the path-length effect [11] the same as in the scanner. The orientation of the film strips with respect to the light source are kept the same as in the scanner by putting the long side of the film strip along the light source, but the glass bed was rotated by 90^0^ to make it stable on the table as the vertical side of it is curved. This change of orientation of the scanner bed (glass) does not have any effect as the orientation of the film with respect to the linear light source remains the same. The film pieces were taped on the scanner bed. A Canon 7D camera was used to take photos with three different lenses of 18 mm, 50 mm and 100 mm focal length. Figure 2 shows the set up for taking photos with the camera. 20 images were taken for each film with each lens, which produced 240 photos (4 film pieces for 4 dose levels × 3 lenses × 20 photos for each film-lens combination). The distances from lens to film strip were 150 mm, 500 mm and 950 mm for 18 mm, 50 mm and 100 mm lenses respectively. These distances were determined by moving the camera back and forth so that the camera captures just the entire film strip, which is 203 mm in the horizontal direction. The images were captured as RAW, which were converted to *.tiff format by using Canon software and then were read and separated into three colour channels in ImageJ V1.49 software.

**Fig. 3:**
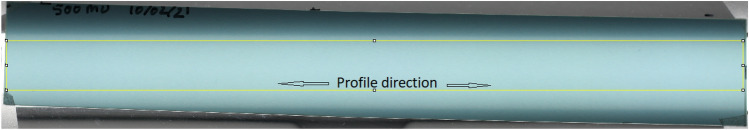
Selection of a rectangle in ImageJ to create a profile along the short side of scanner

In ImageJ an average profile was generated across each film for each combination using a rectangular ROI cropped 1 mm in from the film edge. The average profiles for each colour channel, 20 in total, were analysed in MS Excel where they were normalised to the mean of the central 100 data points (Fig. 3).

Mean and standard deviation of these 20 images for each combination were calculated. The LRA effects were calculated from the profiles as the difference between the maximum (centre) and minimum (edges) values as a percentage, where the values used were the average over 25 data points at the centre and each end respectively. The propagation of uncertainty, which is the standard deviation of normalised pixel values of 20 images, was calculated as root mean square of uncertainty values of maximum and minimum mean percentage value.

$$LRA \,Uncertainty = \sqrt{{Stdv(center)}^{2}+{\mathrm{max}(Stdv\left(left\right), Stdv\left(right\right))}^{2}}$$

## Results

**Fig. 4:**
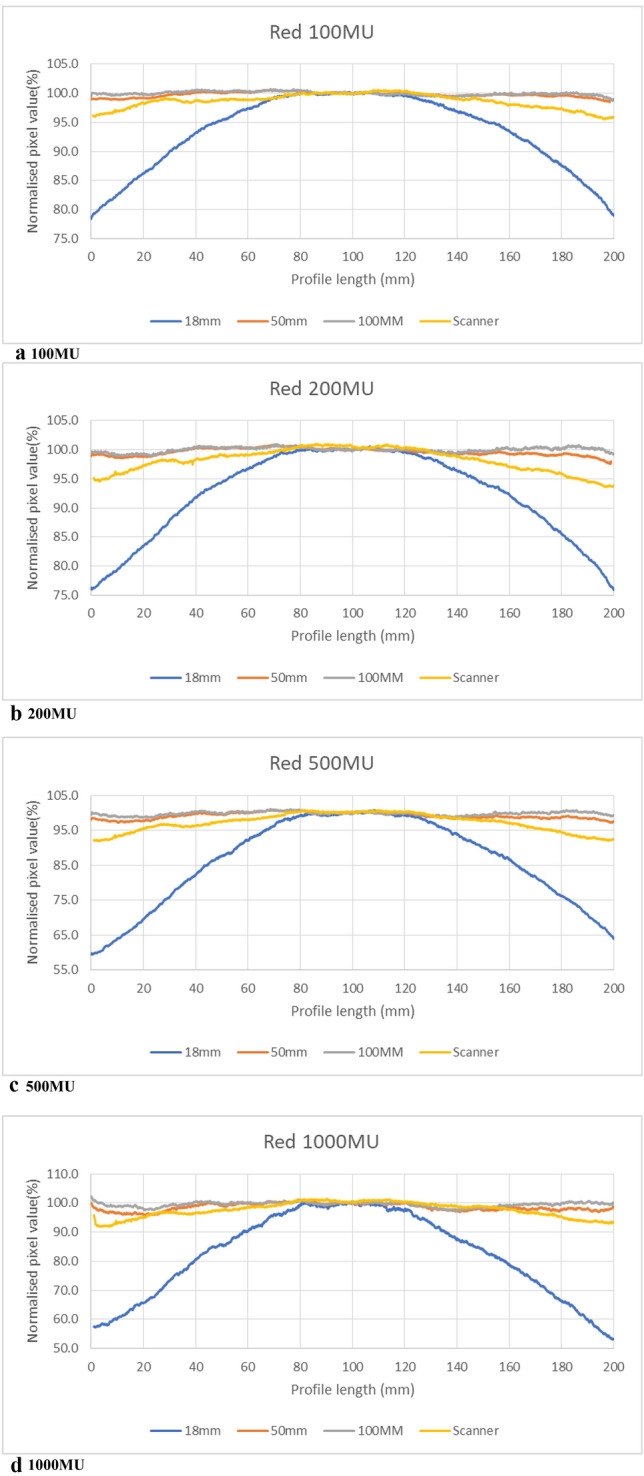
Profiles measured across a strip of EBT3 film that has been exposed at depth in solid water. **a** For 100 MU, **b** for 200 MU, **c** for 500 MU and **d** for 1000 MU. The four profiles in each figure are based on the red channel analysis of images of the film acquired with the Epson V700 flatbed scanner and with a Canon DSLR 7D camera equipped with an 18 mm, 50 mm and 100 mm focal length lens

The Epson V700 has two lenses. The smaller one is for high resolution scanning and the bigger lens is used for the scanning mode used in this work (film with film area guide). The effective focal length of each of the lenses of the Epson V700 scanner was measured to be 38 mm. Figure 4a–d show the profiles in the red channel from films irradiated with 100 MU, 200 MU, 500 MU and 1000 MU respectively (doses to the film as given in the Methods, 1.13 Gy, 2.25 Gy, 5.64 Gy and 11.28 Gy respectively). Each figure shows four profiles, three from images taken with the Canon DSLR camera using 18 mm, 50 mm and 100 mm lenses and the fourth from scanning the films using the Epson V700 scanner, with a lens system of 38 mm focal length. Each profile is for average pixel values from 20 photos and scans. Green channel results were also obtained and showed very similar trends to those of the red channel results. The highest uncertainty as standard deviation of these 20 photos and scans is 1.7% and 1.3% for green channel and red channel respectively.

### LRA effect

Table 1 shows the maximum LRA effect as a maximum percentage difference of edges from the centre of profiles drawn across the film pieces (Figs. 1 and 3) for all the dose levels for all the lenses. Only the results of green and red channels are presented here as no previous studies recommended the use of the blue channel for film dosimetry. A substantial reduction in LRA was seen with the use of the 50 mm and 100 mm lenses, by factors of 3–5 for the 50 mm lens and 4–30 for the 100 mm lens compared to conventional desktop scanner techniques. Using the 100 mm lens, the LRA effect is observed to be 0.3–1.2% at 10 cm out from the centre of the film, with mean values of 0.7% across all four MU values for the green channel and 0.8% for the red. Given the expected + 0.7% in beam flatness in the experimental irradiation conditions at that position (measured using a CC04 ion chamber in exactly the same irradiation conditions and positions), this indicates that the lower normalised pixel values at the edges reflect the higher dose values at that position.

**Table 1:** LRA effect (%) for four lens systems of different focal lengths

Dose level	18 mm		38 mm(scanner)		50 mm		100 mm	
	Green	Red	Green	Red	Green	Red	Green	Red
100 MU	19.1 ± 0.6	21.1 ± 0.5	3.3 ± 0.1	4.1 ± 0.1	1.5 ± 0.2	1.0 ± 0.2	1.0 ± 0.1	1.2 ± 0.2
200 MU	20.6 ± 0.3	23.9 ± 0.3	5.6 ± 0.8	6.9 ± 1.0	2.1 ± 0.2	1.4 ± 0.2	0.7 ± 0.1	0.8 ± 0.1
500 MU	37.0 ± 0.9	40.5 ± 1.0	7.6 ± 0.1	8.2 ± 0.1	1.8 ± 0.3	2.6 ± 0.5	0.9 ± 0.2	0.8 ± 0.3
1000 MU	43.2 ± 0.3	46.6 ± 1.0	8.5 ± 0.2	8.2 ± 0.2	2.4 ± 0.2	2.1 ± 0.4	0.3 ± 0.2	0.4 ± 0.5

## Discussion

The images acquired with different lens systems highlight a systematic variation in the LRA effect with the focal length of the image capturing system. Results show that with a smaller focal length lens system, higher LRA effects are observed. The LRA effect is also observed to increase with dose [9, 10, 16], which is a well-known phenomenon of radiation induced polymerization of active ingredient LiPCDA, which enhances polarization and anisotropic light scattering [9, 21] Schoenfeld et al. [9] investigated the effect of scanner components on LRA effect and stated that the lens system of a flatbed scanner cannot collect all the light scattered from the edges of films. The loss of light collection enhances the optical density towards the edge. The polarization and anisotropic scattering of light caused by the crystals in the active ingredient of the film increases the optical density further at the edge. Wide-angle lenses, i.e. smaller focal length, are used in flatbed scanners to make them compact in size, which creates this loss of light collection from the edges. A bigger focal length lens system needs to be moved further away which results in more scattered light being collected by the lens and a smaller LRA effect.

It is acknowledged that the two systems being evaluated have some differences. Firstly, the light sources used in the scanner and for the camera studies are different. The Epson V700 camera uses a white cold cathode fluorescent light (CCFL) source, whilst a white LED light source was used in the camera work. Larraga-Guiteraze [27] compared the light sources of an Epson V800 and an Epson 11000XL and presented spectra for both, as wavelength versus relative intensity. The Epson V800 uses a white light source and the Epson 11000XL uses a CCFL source and the two are generally representative of the two sources used in this work. Both light sources have the main peak at 550 nm. However, their difference is that the CCFL peak is sharp and has other distinct peaks around the main peak, whilst the spectrum of the LED source is broad, ranging from 470 to 650 nm. The LED source also has another smaller peak at 450 nm. However, if all the peaks are considered, both the light sources have a similar range of wave lengths from 450 to 650 nm.

Also, a flatbed scanner and a DSLR camera work quite differently. As above, in the scanner, light travels from the light source to the scanner’s CCD imager through five mirrors and a lens system [9]. In a DSLR camera, light travels through the lens directly onto the sensor. There is a mirror in the Canon 7D camera, but this moves away from the light path when a picture is taken. As such, both systems are using a transmission style readout with the light source and detector on opposing sides of the film.

In addition, the two systems also utilize different detectors; these being a CCD (Epson scanner) or a CMOS (Camera) detector in the respective systems. The RGB components and quantum efficiency of both systems may vary with respect to wavelength in the Red Green and Blue bandwidths. Although this paper does not analyse or quantify these potential variations, the effects of lens focal length shows similar trends in each bandpass component and as such does not diminish the validity of the trends observed in this work.

**Fig. 5:**
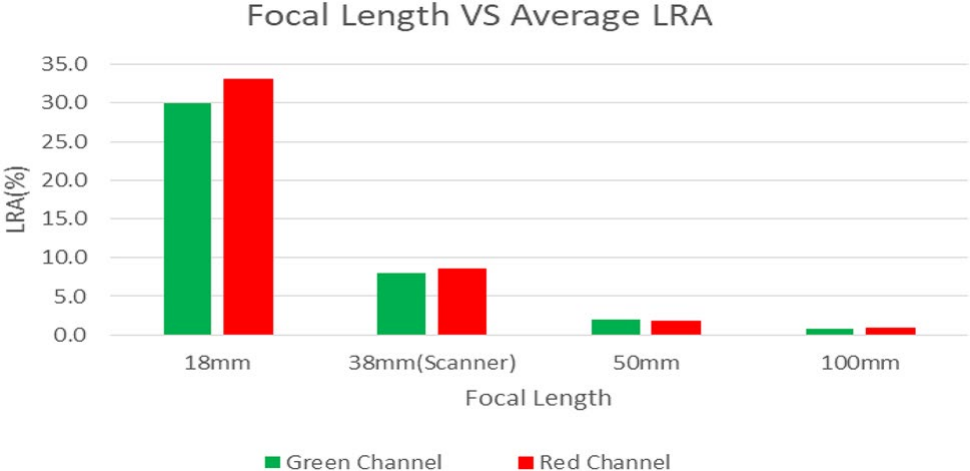
Average LRA of four dose levels of 100 MU, 200 MU, 500 MU and 1000 MU measured from images acquired with an Epson V700 scanner and a Canon 7D camera with three lenses of focal lengths of 18 mm, 50 mm and 100 mm

Figure 5 plots the average LRA of the four dose levels investigated, with respect to the focal lengths of the lens systems used in the study. It shows decreasing LRA for both red and green channels with increasing focal length of the lens systems. It is likely that the current results would be qualitatively similar for other scanners of similar design, but this would need to be confirmed by further studies. Likewise the changes in LRA effect for other types of film would require specific investigation.

The decreased LRA effect using larger focal length imaging systems to analyze films provides an opportunity to create a film scanner system which has the potential to minimize the lateral response artifact. The increase in focal length necessary to capture more light from the edges of the film requires an increased distance between the film and the detection camera. Thus these results highlight the fact that the LRA can be reduced by design choices of specific characteristics of the equipment and imaging system. However, the practicalities of using longer focal length imaging systems are not simple and imply larger systems. Nevertheless, this provides an avenue for further work to explore optimization of new imaging system designs which may be able to remove or at least minimize the LRA effect for Gafchromic EBT3 film. This could potentially reduce or negate the need for scan processing or corrections to scanned raw data for radiochromic film analysis. Specific impacts on dosimetry would depend on various factors, including clinical situation, dose delivered, field sizes/areas irradiated, position off-axis of fields, film type used, etc. and so any novel scanner designs would need careful cost–benefit evaluation on the balance between dosimetric uncertainty gain versus practical system size and use.

## Conclusion

One significant contributing factor to the LRA effect from a flatbed scanner is the lens system. This work shows that utilizing longer focal length lenses can significantly reduce this. For example, if using a 100 mm lens instead of the conventional Epson V700 desktop scanner lens and geometry, the measured LRA was reduced by factors of between 4 and 30, depending on MU (dose) delivered and colour channel analyzed, for doses delivered to Gafchromic EBT3 film of between 1 and 11 Gy. Specifically, the LRA effect was reduced from a range of 4.5 to 11.6% with the scanner to values below 1.2% with the 100 mm lens. As such, an imaging system based on a larger focal length lens could potentially improve the film dosimetry system by reducing the LRA effect and the need for making corrections for it, although this would increase system size.

## Data Availability

All the data are available from the corresponding author by request.
